# Frequency of Loud Snoring and Metabolic Syndrome among Korean Adults: Results from the Health Examinees (HEXA) Study

**DOI:** 10.3390/ijerph14111294

**Published:** 2017-10-26

**Authors:** Claire E. Kim, Sangah Shin, Hwi-Won Lee, Jiyeon Lim, Jong-Koo Lee, Daehee Kang

**Affiliations:** 1 Department of Preventive Medicine, College of Medicine, Seoul National University, 103 Daehakro, Jongnogu, Seoul 03080, Korea; claireekim@snu.ac.kr (C.E.K.); hwiwon@snu.ac.kr (H.-W.L.); jiyeonlim@snu.ac.kr (J.L.); 2 Department of Biomedical Sciences, College of Medicine, Seoul National University, 103 Daehakro, Jongnogu, Seoul 03080, Korea; 3 Department of Food and Nutrition, Chung-Ang University, Gyeonggi-do 17546, Korea; ssa8320@snu.ac.kr; 4 JW Lee Center for Global Medicine, College of Medicine, Seoul National University, IhwaJang-gil 71 Jongno-gu, Seoul 03087, Korea 03087; docmohw@snu.ac.kr; 5 Department of Family Medicine, Seoul National University Hospital, 101 Daehakro, Jongnogu, Seoul 03080, Korea; 6 Institute of Environmental Medicine, Seoul National University Medical Research Center, 103 Daehakro, Jongnogu, Seoul 03080, Korea

**Keywords:** snoring, metabolic syndrome, epidemiology, health examinees study, Korean

## Abstract

Studies regarding the association between snoring and metabolic abnormalities have been inconsistent. We examine whether snoring frequency and obstructive sleep apnea markers are associated with metabolic syndrome (MetS) among Koreans aged 40–69 years. A total of 72,885 subjects (24,856 men, 48,029 women) from the Health Examinees Gem study between 2009 and 2013 were included. Snoring frequency was grouped into five categories (never, 1–3/month, 1–3/week, 4–5/week, 6+/week). Obstructive sleep apnea markers included breathing interruptions and awakenings. Adjusted odds ratios (ORs) and 95% confidence intervals (95% CIs) were calculated through logistic regression. Compared with non-snorers, those who snore 6+/week were associated with increased odds for MetS (OR: 2.07, 95% CI: 1.91–2.25, *p*-trend < 0.0001 among men; OR: 1.45, CI: 1.33–1.58, *p*-trend < 0.0001 among women). Snoring frequency is associated with MetS and its components in both men and women. Snoring and obstructive sleep apnea markers are important indicators of sleep quality, which may facilitate early detection of sleep disorders and further complications such as MetS.

## 1. Introduction

Snoring, a physical phenomenon caused by high-frequency vibrations of the soft palate, pharyngeal walls, the tongue and the epiglottis partially blocking the upper airway, has been increasingly regarded as a pathophysiologic entity that poses risk for adverse health effects [1]. Specifically, it has been identified as an important indicator of sleep quality and consequentially as a potential marker for sleep disorders such as obstructive sleep apnea (OSA) [2,3]. Accordingly, validated tools measuring OSA, such as the Basic Nordic Sleep Questionnaire, pose several questions that aim to assess “habitual snoring” [4,5].

Despite the growing number of snoring studies, methodological differences such as the categorization of snoring frequency and varying OSA-related questions have produced mixed results. Studies conducted in Western and Asian countries on snoring have tended to dichotomize the exposure to be the presence of “habitual” snoring with no consensus on the number of snoring accounts per week [6,7]. Furthermore, few studies assessed the effects of breathing interruptions and awakenings, both of which are common symptoms of OSA [4,8,9]. Examination of dose–response snoring frequency with finer categories as well as the other OSA markers may improve clinical interviews and epidemiological questionnaires in predicting sleep-related health outcomes. Moreover, self-report questionnaires may be a low-cost, noninvasive tool for large epidemiological studies to screen the general population for people who may be unknowingly at risk for OSA and its subsequent health outcomes [5,10,11].

Metabolic syndrome (MetS) is characterized by a cluster of cardiovascular risk factors such as elevated waist circumference, high triglyceride levels, low high-density cholesterol levels, hypertension, and high fasting glucose. Previous observational studies suggest that snoring is associated with MetS and its components [12,13,14,15] including dyslipidemia [16], hypertension [17], and diabetes [6]. Additionally, prospective studies have identified snoring as an independent risk factor for MetS [18]. Given the high prevalence of MetS among Korean adults [19], identifying modifiable risk factors such as snoring is warranted. Additionally, the prevalence of snoring and OSA among the Korean population is similar to those of Western societies, despite the overall lower average BMI among Koreans. Therefore, studying the relationship between snoring and MetS among Koreans may provide insight into the non-obesity related risk factors contributing to the pathogenesis of MetS [20].

The current study aimed to examine snoring frequency, breathing interruptions, and awakenings with the odds for MetS and its components. To consider the gender differences in the prevalence of snoring [7], we explored whether the studied relationships differed between men and women. We also performed a subgroup analysis by normal and overweight BMI to address potential confounding by BMI. To our knowledge, this is the largest study assessing the gender stratified, dose–response relationship of snoring frequency and OSA markers with MetS and its components.

## 2. Materials and Methods

### 2.1. Study Population

**Figure 1:**
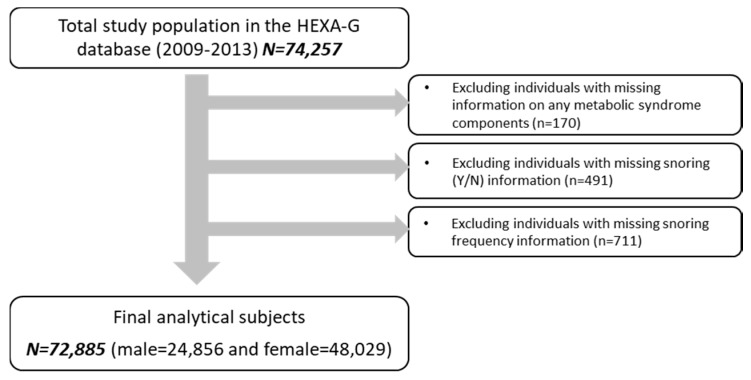
Flow diagram of analytical sample, the HEXA Gem (HEXA-G) 2009–2013.

The Health Examinees (HEXA) study is a large-scale community-based genomic survey conducted in Korea 2004–2013 of adults aged 40–69. Description of the HEXA study rationale, study design, and baseline characteristics can be found in its introductory papers [21,22]. Updated from the previously published HEXA studies, the current study uses the HEXA Gem (HEXA-G) participant sample which includes additional eligibility criteria on the participating sites (i.e., health examination centers and training hospitals) and is outlined in detail in previous literature [23]. In this study, HEXA-G data from years 2009–2013 were used as prior surveys did not include snoring-related questions. Of the eligible HEXA-G (*n* = 74,257) study subjects, we excluded those without information on any of the MetS components (*n* = 170): snoring (Yes/No) (*n* = 491) or snoring frequency (*n* = 711). The final analytic sample included a total of 72,885 subjects with 24,856 men and 48,029 women (Figure 1). The HEXA study was approved by the Ethics Committee of the Korean Health and the institutional review boards of all participating hospitals (IRB No. E-1503-103-657). All study participants provided informed consent prior to entering the study.

### 2.2. Assessment of Snoring Frequency and Symptoms

Presence of snoring was assessed with the following question: “I have been told that I snore loudly” with a Yes or No response. For those who answered “Yes”, a follow-up question regarding snoring frequency was asked: “How often do you snore?” with the available five category responses: very rarely, 1–3/month, 1–3/week, 4–5/week, 6+/week. In the current study, participants who answered, “No” to the presence of snoring and “Very rarely” to the snoring frequency question were grouped into the “Never” category and served as the reference group for the final analysis.

Breathing interruptions were assessed with a Yes or No question: “I have been told (by someone who shares the room with me) that I’ve displayed breathing interruptions while snoring”. Awakenings were assessed with a Yes or No question: “There were times in which I have woken up from snoring”. In the final analysis, for both snoring symptoms, the “No” category, which includes both non-snorers and snorers without the OSA markers, was set as the reference group. This reference group allowed the evaluation of each OSA marker “independently” in its association with MetS and its components. As these markers are highly correlated, snoring frequency and presence of awakenings (for breathing interruptions, vice versa) were not adjusted in the final model.

### 2.3. Definition of Metabolic Syndrome (MetS)

The current study defined MetS using the National Cholesterol Education Program Adult Treatment Panel III [24]. Participants who met three or more of the following criteria were classified as having MetS: (1) waist circumference (WC) ≥ 90 and ≥80 cm for men and women, respectively; (2) triglycerides (TG) ≥ 150 mg/dL or drug treatment for elevated triglycerides; (3) high-density lipoprotein cholesterol (HDL-C) ≤ 40 and ≤50 mg/dL in men and women, respectively; (4) systolic blood pressure (BP) ≥ 130, diastolic BP ≥ 85 mmHg or drug treatment for elevated BP; and (5) fasting glucose ≥ 100 mg/dL or drug treatment for elevated fasting glucose.

### 2.4. Covariates

Sociodemographic factors such as age (continuous), BMI (continuous), education, occupation, marital status, and menopausal status (women only) were assessed. Education was categorized as middle school or below, high school graduate, and college or above. Occupation was categorized as non-manual, manual, and unemployed. Marital status was categorized into married or single (including never married, separated, divorced, or bereaved). Menopausal status was classified as either post-menopausal women who have gone a year without menstrual flow or pre-menopausal women who currently experience monthly menstrual cycles.

Other covariates included lifestyle factors such as sleep duration, smoking, alcohol drinking, and regular exercise. Sleep duration represented the average amount of sleep per day, a continuous variable measured in minutes. Smoking status was ascertained by posing the following question: “Have you smoked more than 5 packs of cigarettes (100 cigarettes) in your lifetime?” People who responded as never having smoked 100 cigarettes were defined as non-smokers; subjects who had smoked ≥100 cigarettes during their lifetime but did not smoke at the time of the survey were classified as past smokers; subjects who had smoked ≥100 cigarettes during their lifetime and still smoked cigarettes at the time of the survey were classified as current smokers. Alcohol drinking status was determined by the following question: “Are you unable to consume alcohol or do you refuse to do so (for religious reasons, etc.)?” Respondents who answered “yes” were determined as non-drinkers. Drinkers who have answered the question, “Do you still drink” with “no” were also categorized as non-drinkers and with “yes” as current drinkers. Exercise was assessed by the question: “Do you participate in any sport regularly (enough to sweat)? Respondents who answered “no” were assigned to the non-regular exercise group; subjects who responded “yes” were assigned to the regular exercise group. For all covariates, missing data was categorized as “unknown”.

### 2.5. Statistical Analysis

All estimates were calculated separately by gender to investigate the gender differences in the association between snoring frequency and symptoms and MetS. Gender interaction *p*-values were assessed by the likelihood ratio tests with the use of a cross-product term. A chi-square test (for categorical variables) and ANOVA (for continuous variables) were performed to analyze the basic characteristics of the study population with respect to snoring categories. All variables were tested for normality using the skewness-kurtosis test. For the main OR analyses, an adjusted model including age (continuous), BMI (continuous; not adjusted for WC OR), education (≤middle school, high school, ≥college, unknown), occupation (manual, non-manual, unemployed, unknown), marital status (married, single, unknown), menopausal status (pre-, post-, unknown; women only), smoking (never, past, current, unknown; men only), alcohol drinking (current, non, unknown), regular exercise (yes, no unknown), and sleep duration (continuous) as covariates was used. Smoking was excluded from the model for women because the prevalence of smoking (~6%) was very low among all snoring categories. As BMI is a modifier of the association between snoring and MetS, BMI was adjusted in the final model. However, since BMI is highly correlated with waist circumference, the final model excluded BMI from its WC OR calculation. Moreover, to account for comorbidities, a sensitivity analysis by excluding subjects with a history of type 2 diabetes, hypertension, and dyslipidemia was performed. Supplementary analyses involving BMI subgroups and number of OSA markers (i.e., snoring, breathing interruptions, awakenings) on the odds for MetS were also conducted.

Multivariable logistic regression models were used to calculate prevalence odds ratios (ORs) and 95% confidence intervals (95% CIs). We tested the linear trend across snoring frequency using general linear regression. All of the *p*-values were two-sided, and statistical significance was set at below 0.05. All statistical analyses were conducted using the SAS software version 9.4 (SAS Institute, Cary, NC, USA).

## 3. Results

Baseline characteristics of the study population by snoring frequency are summarized in Table 1. About 14.6% of men and 7.3% of women indicated snoring 6+/week. All selected covariates for the final analysis, with the exception of occupation in men and alcohol drinking in women, differed with statistical significance across the snoring frequency categories.

The overall prevalence of MetS was 29.3% in men and 24.9% in women (Table 2). There were significant differences in the prevalence of MetS and all five of its components based on snoring frequency in both men and women (MetS and all five components *p* < 0.0001). The odds ratios (ORs) and 95% confidence interval (95% CIs) for MetS and its components with snoring categories are presented in Table 3. Using the “never” snoring frequency category as a reference, snoring 6+/week was associated with statistically significant increased odds for MetS in both men and women (OR: 2.07, 95% CI: 1.91–2.25, *p*-trend < 0.0001 among men; OR: 1.45, 95% CI: 1.33–1.58, *p*-trend < 0.0001 among women) when adjusted for age, BMI, education, occupation, marital status, smoking (men only), alcohol drinking, regular exercise, menopausal status (women only), and sleep duration.

Furthermore, in both men and women, snoring 6+/week was associated with increased adjusted (excluding BMI in WC calculation) ORs of elevated waist circumference, hypertriglyceridemia, low HDL-C, elevated blood pressure, and high fasting glucose. Gender differences were observed in the ORs for MetS and its components (all gender *p*-interaction value < 0.0001).

Excluding subjects with either a history of type 2 diabetes, hypertension, or dyslipidemia did not affect the association of snoring frequency with the odds for MetS (OR: 2.12, 95% CI: 1.90–2.37, *p*-trend < 0.0001 in men and OR: 2.16, 95% CI: 1.94–2.40, *p*-trend < 0.0001 in women). Moreover, in the subgroup analysis stratified by BMI group, snoring frequency was significantly associated with increased ORs for MetS in both normal and overweight BMI groups (Table S1; BMI < 25 kg/m^2^, OR: 1.26, 95% CI: 1.09–1.45 and ≥25 kg/m^2^, OR: 1.59, 95% CI: 1.41–1.78 among men; BMI < 25 kg/m^2^, OR: 1.79, 95% CI: 1.58–2.01 and ≥25 kg/m^2^, OR: 1.67, 95% CI: 1.49–1.87 among women). As BMI is a modifier of the association between snoring and MetS, BMI was adjusted in the final model (with the exception of OR calculation for WC).

The adjusted ORs and CIs for OSA symptoms, breathing interruptions and awakenings, are listed in Table 4. Among 24,783 men who answered the breathing interruption question, 4062 (16.4%) experienced breathing interruptions; among 47,927 women, 1596 (3.3%) experienced breathing interruptions. Among 24,824 men who answered the awakening question, 2999 (12.1%) experienced awakenings; among 47,991 women, 6226 (13.0%) experienced awakenings. Breathing interruptions were associated with greater odds of MetS (OR: 1.58, 95% CI: 1.47–1.70 among men; OR: 1.32, 95% CI: 1.18–1.49 among women), elevated waist circumference (men only), hypertriglyceridemia, low HDL-C (women only), elevated BP, and high fasting glucose. Awakenings were associated with greater odds of MetS (OR: 1.39, 95% CI: 1.28–1.50 among men; OR: 1.21, 95% CI: 1.13–1.29), elevated waist circumference (among men), hypertriglyceridemia, low HDL-C (women only), elevated BP, and high fasting glucose were observed.

In another supplementary analysis (Table S2) examining the association between the number of OSA markers and MetS, greater presence of OSA markers was associate with increased OR for MetS (OR: 2.03, 95% CI: 1.81–2.27, *p*-trend < 0.0001 among men; OR: 1.48, 95% CI: 1.28–1.72, *p*-trend < 0.0001). 

## 4. Discussion

In the current study, snoring frequency was associated with increased odds of MetS and its components (elevated waist circumference, hypertriglyceridemia, reduced HDL-C, elevated BP, and high fasting glucose) among Korean adults. Additionally, breathing interruptions had greater odds of MetS, elevated waist circumference (men only), hypertriglyceridemia, low HDL-C (women only), elevated BP, and high fasting glucose. Awakenings were associated with greater odds of MetS and its components in both men and women, with the exception of low HDL-C in men and elevated waist circumference in women.

Given that the association between snoring and OSA markers on MetS is multifactorial, there are several important covariates to consider. Gender differences may be due to upper airway anatomy and physiology such as pharyngeal collapsibility and central respiratory drive [25]. Another study reported that female sex hormones may be protective against sleep-disordered breathing, as postmenopausal women had a higher risk of sleep-disordered breathing than either premenopausal women or postmenopausal women on hormone replacement therapy [26]. Even adjusting for menopausal status, however, gender differences were observed with men having greater odds of MetS and its components by increasing snoring frequency than in women. Furthermore, while the underlying mechanisms of upper airway narrowing are influenced by multiple factors, studies have reported that obesity increases fat deposits, which may affect the metabolic activity of pharyngeal adipose tissue [27,28]. To this regard, we performed a subgroup analysis with the following stratified groups: normal (<25 kg/m^2^) and overweight/obese (≥25 kg/m^2^) BMI. An increasing trend of MetS and its components were observed in both BMI groups (Table S1). To account for the differences in OR magnitude, BMI was adjusted in the final analysis. Of note, the prevalence of snoring and OSA in the current population is similar to those of Western societies, despite the overall lower average BMI. This suggests that a non-obesity related risk factor may contribute to the pathogenesis of the disease [20]. Lastly, alcohol has been found to increase the pharynx collapsibility via the genioglossal and palatine tensor muscles [29] and therefore was controlled in the final model.

The clinical significance of snoring without diagnosed OSA (hereafter termed “simple snoring”) has been controversial. While snoring is one of the main manifestations of OSA, other markers of OSA such as breathing interruptions and awakenings may be a significant contributor more or less to the subsequent health outcomes. Previous studies assessing snoring on the risk of cardiovascular disease (CVD), coronary heart disease (CHD), and its risk factors have noted the caveat that observed negative effects of snoring may be confounded by the presence of OSA [30]. A study in the US reported that snoring was associated with CVD only when it was paired with daytime sleepiness, a symptom of OSA [31]. Another recent study in Finland suggested that, while OSA and snoring related to sleep-disordered breathing are associated with cardiovascular risk, simple snoring is not [10]. Furthermore, a study in Australia using a home sleep apnea monitoring devise concluded that there is no measure of simple snoring associated with all-cause mortality, incident cardiovascular disease or stroke [32]. In contrast, a US study using a polysomnography concluded that simple snoring is associated with carotid artery atherosclerosis [33]. Another recent US study, using a home sleep test, reported that even after adjusting for variables such as BMI and OSA severity, increased simple snoring was associated with increased all-cause mortality [34]. These varying results suggest that snoring with or without OSA merits attention and highlights the need for objective measurements to more accurately quantify snoring frequency and other OSA markers in order to better understand its association with health consequences [35].

There has been less documentation on the association between snoring and MetS. While the biological mechanisms regarding snoring and its effect on MetS is yet uncertain, there are several plausible explanations for the phenomenon. Snoring and the consequent pauses in breathing have been found to increase the sympathetic nervous system [36] and to increase oxidative stress [37,38]. This, in turn, impairs glucose homeostasis and increases glycogenesis and gluconeogenesis leading to insulin resistance [39,40]. Further, a study reported raised arterial pressure and respiratory difficulty, which may potentially be a result of increasing micro-arousals, sleep fragmentation, and disrupted restorative value of sleep [14]. Intermittent hypoxia and arousals caused by snoring may also cause a drop in blood pressure, which leads to an increased sympathetic tone, which is involved in the development pulmonary hypertension [41]. Another study found that snoring was linked to a persistent increase in urinary albumin, a risk factor for diabetes [42]. Others observed elevated hemoglobin levels, which has been studied to be a risk factor for all components of MetS [43,44].

While our results display an association between snoring frequency and MetS, there are limitations to consider. First, this is a cross-sectional study, so causality between snoring and MetS cannot be assessed. Future studies using the total HEXA-G sample on sleep duration and subsequent risk of MetS are warranted to confirm these preliminary findings. Second, the questions used to assess snoring frequency and symptoms were based on self-report questionnaire rather than objective measures via polysomnography, allowing for possible misclassification. Nevertheless, self-reported sleep questionnaires are a useful preventative tool in large epidemiological studies [5]. Third, OSA was not measured, so health effects of simple snoring cannot be determined. Finally, while the current study adjusted for all relevant covariates, there may have been residual confounders that were unmeasured. Regardless of these limitations, this is the largest study examining gender stratified, dose-response snoring frequency as well as other markers of OSA with MetS and its components. Specifically, in Korea, snoring-focused literature has been scarce. The association between snoring and MetS among Koreans, who have relatively low average BMI, highlights the need for greater research on the role of non-obesity factors on MetS development.

## 5. Conclusions

Taken together, our results suggest that greater snoring frequency, breathing interruptions and awakenings are associated with MetS and its components. In clinical practice, screening of individuals at risk for OSA should focus on not only the presence of snoring but also other markers of OSA that indicate its intensity. Additionally, we underline the need for prospective studies to examine the association between snoring and MetS with more objective measures to establish causality. Assessing snoring and OSA markers comprehensively may improve sleep quality and also allow for early recognition of sleep disorders and accompanying health consequences such as MetS.

## Figures and Tables

**Table 1:** Baseline characteristics ^a^ by snoring frequency, the Health Examinees Gem (HEXA-G) 2009–2013.

	Snoring Frequency						
	Total	Never	1–3/month	1–3/week	4–5/week	6+/week	*p*-Value ^b^
*Men, n*	*24856*	*9555 (38.4)*	*4857 (19.5)*	*5190 (20.9)*	*1634 (6.6)*	*3620 (14.6)*	
Age, years	53.8 ± 8.5	53.6 ± 8.8	53.7 ± 8.4	53.5 ± 8.2	54.9 ± 8.1	53.9 ± 8.4	<0.0001
BMI, kg/m^2^	24.4 ± 2.8	23.7 ± 2.6	24.4 ± 2.6	24.8 ± 2.7	25.1 ± 2.8	25.5 ± 2.9	<0.0001
Current smokers	18642 (75.0)	6916 (72.4)	3573 (73.6)	4069 (78.4)	1274 (78.0)	2810 (77.6)	<0.0001
Current drinkers	18219 (73.3)	6651 (69.6)	3577 (73.7)	4061 (78.3)	1245 (76.2)	2685 (74.2)	<0.0001
Regular exercisers	14407 (58.0)	5398 (56.5)	2939 (60.5)	3127 (60.3)	910 (55.7)	2033 (56.2)	<0.0001
College or above	9316 (37.5)	3540 (37.1)	1878 (38.7)	1999 (38.5)	572 (35.0)	1327 (36.7)	0.0276
Manual occupation	11,964 (48.1)	4571 (47.8)	2360 (48.6)	2473 (47.7)	777 (47.6)	1783 (49.3)	0.0701
Married	23,180 (93.3)	8686 (90.9)	4585 (94.4)	4934 (95.1)	1551 (94.9)	3424 (94.6)	<0.0001
Sleep duration, hours	6.9 ± 1.2	6.9 ± 1.2	6.9 ± 1.1	6.9 ± 1.1	6.9 ± 1.1	6.9 ± 1.2	0.0091
*Women, n*	*48,029*	*25,590 (53.3)*	*10,420 (21.7)*	*6736 (14.0)*	*1779 (3.7)*	*3504 (7.3)*	
Age, years	52.6 ± 7.8	51.5 ± 7.9	53.0 ± 7.5	54.2 ± 7.5	54.7 ± 7.4	55.1 ± 7.4	<0.0001
BMI, kg/m^2^	23.6 ± 3.0	23.0 ± 2.7	23.8 ± 2.9	24.3 ± 3.1	24.7 ± 3.3	25.3 ± 3.3	<0.0001
Current smokers	1875 (3.9)	942 (3.7)	380 (3.7)	288 (4.3)	76 (4.3)	189 (5.4)	<0.0001
Current drinkers	15,522 (32.3)	8229 (32.2)	3437 (33.0)	2224 (33.0)	558 (31.4)	1074 (30.7)	0.0676
Regular exercisers	24,826 (51.7)	13,026 (50.9)	5691 (54.6)	3489 (51.8)	886 (49.8)	1734 (49.5)	<0.0001
College or above	10,065 (21.0)	6197 (24.2)	2031 (19.5)	1109 (16.5)	267 (15.0)	461 (13.2)	<0.0001
Manual occupation	13,046 (27.2)	6706 (26.2)	2787 (26.8)	1948 (28.9)	533 (30.0)	1072 (30.6)	<0.0001
Married	41,688 (86.8)	22,112 (86.4)	9205 (88.3)	5861 (87.0)	1539 (86.5)	2971 (84.8)	<0.0001
Post-menopausal	28,239 (58.8)	13,504 (52.8)	6455 (62.0)	4551 (67.6)	1243 (69.9)	2486 (71.0)	<0.0001
Sleep duration, hours	6.9 ± 1.2	6.9 ± 1.2	6.9 ± 1.2	6.9 ± 1.2	6.9 ± 1.2	6.9 ± 1.3	0.0079

^a^ Values are *n* (%) or mean ± SD. ^b^ *p*-values for differences among snoring categories were calculated by chi-square tests for categorical variables and ANOVA for continuous variables.

**Table 2:** Metabolic Syndrome (MetS) ^a^ prevalent cases ^b^ by snoring frequency, the Health Examinees Gem (HEXA-G) 2009–2013.

	Snoring Frequency						
	Total	Never	1–3/month	1–3/week	4–5/week	6+/week	*p*-Value ^c^
*Men, n*	*24,856*	*9555*	*4857*	*5190*	*1634*	*3620*	
MetS	7276 (29.3)	2216 (23.2)	1355 (27.9)	1709 (32.9)	598 (36.6)	1398 (38.6)	<0.0001
WC ≥ 90 cm	6911 (27.8)	1990 (20.8)	1305 (26.9)	1603 (30.9)	546 (33.4)	1467 (40.5)	<0.0001
Serum TG ≥ 150 mg/dL	10,077 (40.5)	3428 (35.9)	1923 (39.6)	2299 (44.3)	729 (44.6)	1698 (46.9)	<0.0001
Serum HDL-C ≤ 40 mg/dL	5793 (23.3)	2011 (21.1)	1101 (22.7)	1207 (23.3)	429 (26.3)	1045 (28.9)	<0.0001
BP ≥ 130/85 mmHg	13,323 (53.6)	4613 (48.3)	2582 (53.2)	2982 (57.5)	990 (60.6)	2156 (59.6)	<0.0001
Fasting glucose ≥ 100 mg/dL	8517 (34.3)	2998 (31.4)	1648 (33.9)	1879 (36.2)	637 (39.0)	1355 (37.4)	<0.0001
*Women, n*	*48,029*	*25,590*	*104,20*	*6736*	*1779*	*3504*	
MetS	11,980 (24.9)	4874 (19.1)	2796 (26.8)	2205 (32.7)	657 (36.9)	1448 (41.3)	<0.0001
WC ≥ 80 cm	19,121 (39.8)	8172 (31.9)	4538 (43.6)	3313 (49.2)	934 (52.5)	2164 (61.8)	<0.0001
Serum TG ≥ 150 mg/dL	11,730 (24.4)	5288 (20.7)	2656 (25.5)	2020 (30.0)	567 (31.9)	1199 (34.2)	<0.0001
Serum HDL-C ≤ 50 mg/dL	17,047 (35.5)	8206 (32.1)	3872 (37.2)	2637 (39.2)	740 (41.6)	1592 (45.4)	<0.0001
BP ≥ 130/85 mmHg	18,380 (38.3)	8249 (32.2)	4280 (41.1)	3148 (46.7)	882 (49.6)	1821 (52.0)	<0.0001
Fasting glucose ≥ 100 mg/dL	9330 (19.4)	4127 (16.1)	2128 (20.4)	1602 (23.8)	474 (26.6)	999 (28.5)	<0.0001

^a^ MetS: the presence of 3 or more of the following components: (1) elevated waist circumference (WC); (2) high triglyceride (TG) levels; (3) low high density lipoprotein-cholesterol (HDL-C) level or taking anticholesterol medication; (4) high blood pressure (BP) or taking antihypertensive medicine; (5) high fasting glucose levels or taking medication to treat diabetes mellitus. ^b^ Values are *n* (%). ^c^ *p*-values for differences among snoring categories were calculated by chi-square tests.

**Table 3:** Odds ratio (ORs) ^a^ of metabolic syndrome (MetS) ^b^ by snoring frequency, the Health Examinees Gem (HEXA-G) 2009–2013.

	Snoring Frequency					
	Never	1–3/month	1–3/week	4–5/week	6+/week	*p*-Trend ^c^
*Men n = 24,856 ^d^*	*9555*	*4857*	*5190*	*1634*	*3620*	
MetS	Ref.	1.29 (1.19–1.40)	1.63 (1.51–1.75)	1.85 (1.65–2.07)	2.07 (1.91–2.25)	<0.0001
WC ≥ 90 cm	Ref.	1.40 (1.29–1.52)	1.69 (1.57–1.83)	1.86 (1.66–2.09)	2.57 (2.37–2.79)	<0.0001
Serum TG ≥ 150 mg/dL	Ref.	1.18 (1.09–1.26)	1.39 (1.30–1.49)	1.44 (1.29–1.60)	1.56 (1.45–1.69)	<0.0001
Serum HDL-C ≤ 40 mg/dL	Ref.	1.04 (0.95–1.13)	1.04 (0.96–1.13)	1.12 (0.99–1.27)	1.22 (1.11–1.34)	<0.0001
BP ≥ 130/85 mmHg	Ref.	1.21 (1.13–1.30)	1.44 (1.35–1.55)	1.57 (1.41–1.75)	1.57 (1.45–1.70)	<0.0001
Fasting glucose ≥ 100 mg/dL	Ref.	1.12 (1.04–1.21)	1.23 (1.14–1.32)	1.32 (1.19–1.48)	1.29 (1.19–1.40)	<0.0001
*Women n = 48,029 ^d^*	*25,590*	*10,420*	*6736*	*1779*	*3504*	
MetS	Ref.	1.22 (1.15–1.29)	1.34 (1.25–1.44)	1.40 (1.25–1.57)	1.45 (1.33–1.58)	<0.0001
WC ≥ 80 cm	Ref.	1.55 (1.48–1.63)	1.81 (1.71–1.92)	1.98 (1.79–2.19)	2.87 (2.66–3.09)	<0.0001
Serum TG ≥ 150 mg/dL	Ref.	1.12 (1.06–1.18)	1.25 (1.17–1.33)	1.25 (1.12–1.39)	1.27 (1.17–1.38)	<0.0001
Serum HDL-C ≤ 50 mg/dL	Ref.	1.10 (1.05–1.16)	1.09 (1.02–1.15)	1.11 (1.00–1.23)	1.21 (1.12–1.30)	<0.0001
BP ≥ 130/85 mmHg	Ref.	1.21 (1.15–1.27)	1.31 (1.24–1.39)	1.34 (1.20–1.48)	1.32 (1.22–1.43)	<0.0001
Fasting glucose ≥ 100 mg/dL	Ref.	1.13 (1.07–1.21)	1.23 (1.14–1.31)	1.32 (1.18–1.48)	1.33 (1.22–1.45)	<0.0001

^a^ ORs adjusted for: age (continuous), BMI (continuous), education (≤middle school, high school, ≥college, unknown), occupation (manual, non-manual, unemployed, unknown), marital status (married, single, unknown), smoking (current, past, never, unknown; men only), alcohol drinking (current, non-, unknown), regular exercise (yes, no, unknown), menopausal status (pre-, post-, unknown; women only), and sleep duration (continuous). ^b^ MetS: the presence of 3 or more of the following components: (1) elevated waist circumference (WC); (2) high triglyceride (TG) levels; (3) low high density lipoprotein–cholesterol (HDL-C) or taking anticholesterol medication; (4) high blood pressure (BP) or taking antihypertensive medicine; (5) high fasting glucose levels or taking medication to treat diabetes mellitus. ^c^ Linear trends across snoring frequency categories were calculated by general linear regression. ^d^ Gender *p*-interaction value < 0.0001; interaction term was assessed by likelihood ratio tests with the use of a cross-product term

**Table 4:** Odds ratio (ORs) ^a^ of metabolic syndrome (MetS) ^b^ by OSA markers, the Health Examinees Gem (HEXA-G) 2009–2013.

**Presence of Breathing Interruptions**		
	*Men (n = 4062)*	*Women ^c^ (n = 1596)*
MetS	1.58 (1.47–1.70)	1.32 (1.18–1.49)
WC ≥ 90 cm (M)/≥80 cm (W)	1.72 (1.60–1.84)	1.06 (0.92–1.21)
Serum TG ≥ 150 mg/dL	1.28 (1.19–1.37)	1.28 (1.15–1.43)
Serum HDL-C ≤ 40 mg/dL (M)/≤50 mg/dL (W)	1.07 (0.99–1.16)	1.18 (1.06–1.31)
BP ≥ 130/85 mmHg	1.40 (1.31–1.51)	1.30 (1.17–1.45)
Fasting glucose ≥ 100 mg/dL	1.22 (1.13–1.31)	1.26 (1.12–1.41)
**Presence of Awakenings**		
	*Men (n = 2999)*	*Women ^c^ (n = 6226)*
MetS	1.39 (1.28–1.50)	1.21 (1.13–1.29)
WC ≥ 90 cm (M)/≥80 cm (W)	1.48 (1.37–1.61)	0.99 (0.90–1.07)
Serum TG ≥ 150 mg/dL	1.24 (1.15–1.34)	1.17 (1.10–1.25)
Serum HDL-C ≤ 40 mg/dL (M)/≤50 mg/dL (W)	0.99 (0.91–1.09)	1.08 (1.00–1.15)
BP ≥ 130/85 mmHg	1.28 (1.18–1.38)	1.19 (1.10–1.26)
Fasting glucose ≥ 100 mg/dL	1.16 (1.07–1.25)	1.18 (1.10–1.26)

^a^ ORs adjusted for: age (continuous), BMI (continuous), education (≤middle school, high school, ≥college, unknown), occupation (manual, non-manual, unemployed, unknown), marital status (married, single, unknown), smoking (current, past, never, unknown; men only), alcohol drinking (current, non-, unknown), regular exercise (yes, no, unknown), menopausal status (pre-, post-, unknown; women only), and sleep duration (continuous). ^b^ MetS: the presence of 3 or more of the following components: (1) elevated waist circumference (WC); (2) high triglyceride (TG) levels; (3) low high density lipoprotein-cholesterol (HDL-C) or taking anticholesterol medication; (4) high blood pressure (BP) or taking antihypertensive medicine; (5) high fasting glucose levels or taking medication to treat diabetes mellitus. ^c^ Gender *p*-interaction value < 0.0001; interaction term was assessed by likelihood ratio tests with the use of a cross-product term.

## Supplementary Materials

The following are available online at www.mdpi.com/1660-4601/14/11/1294/s1, Table S1: Odds ratio (ORs) of metabolic syndrome (MetS) by snoring frequency stratified by BMI, the Health Examinees Gem (HEXA-G) 2009–2013, Table S2: Odds ratio (ORs) of metabolic syndrome (MetS) by number of obstructive sleep apnea (OSA) markers, the Health Examinees-Gem (HEXA-G) 2009–2013.
