# Supplementary material for: Frequency of Loud Snoring and Metabolic Syndrome among Korean Adults: Results from the Health Examinees (HEXA) Study
Source: Int J Environ Res Public Health. 2017 Oct 26;14(11):1294. doi: 10.3390/ijerph14111294 (PMC5707933; doi:10.3390/ijerph14111294)
Supplement: Supplementary file 1 [file ijerph-14-01294-s001.pdf]

International Journal of
Environmental Research and Public Health

MDPI

Supplementary Materials: Frequency of Loud Snoring and Metabolic Syndrome among Korean Adults: Results from the Health Examinees (HEXA) Study

Claire E. Kim, Sangah Shin, Hwi-Won Lee, Jiyeon Lim, Jong-koo Lee, and Daehee Kang

Table S1. Odds ratio (ORs) [a] of metabolic syndrome (MetS) [b] by snoring frequency stratified by BMI, the Health Examinees Gem (HEXA-G) 2009–2013.

	Snoring Frequency						
	**Never**	**1–3 times/mth**	**1–3 times/wk**	**4–5 times/wk**	**6+ times/wk**	***p*-Trend** [c]	***p*-Interaction** [d]
Men							
BMI < 25 kg/m² n = 14918	*6694*	*2926*	*2843*	*828*	*1627*		
MetS	Ref.	1.20 (1.07–1.36)	1.38 (1.23–1.55)	1.46 (1.21–1.75)	1.26 (1.09–1.45)	<0.0001	0.0059
WC ≥ 90 cm	Ref.	1.25 (1.06–1.47)	1.17 (0.99–1.38)	1.03 (0.78–1.36)	1.56 (1.29–1.88)	<0.0001	0.2056
Serum TG ≥ 150 mg/dL	Ref.	1.08 (0.98–1.18)	1.23 (1.12–1.35)	1.27 (1.09–1.48)	1.26 (1.13–1.42)	<0.0001	0.9157
Serum HDL-C ≤ 40 mg/dL	Ref.	1.14 (1.02–1.28)	1.12 (1.00–1.26)	1.30 (1.09–1.56)	1.35 (1.18–1.55)	<0.0001	0.3967
BP ≥ 130/85 mmHg	Ref.	1.18 (1.08–1.29)	1.27 (1.16–1.39)	1.33 (1.15–1.54)	1.20 (1.08–1.34)	<0.0001	0.1045
Fasting glucose ≥ 100 mg/dL	Ref.	1.05 (0.95–1.16)	1.17 (1.06–1.29)	1.12 (0.96–1.32)	0.97 (0.86–1.10)	0.2596	0.0067
BMI ≥ 25kg/m² n = 9938	*2861*	*1931*	*2347*	*806*	*1993*		
MetS	Ref.	1.05 (0.93–1.18)	1.28 (1.14–1.43)	1.39 (1.19–1.63)	1.59 (1.41–1.78)	<0.0001	
WC ≥ 90 cm	Ref.	1.05 (0.94–1.19)	1.23 (1.10–1.38)	1.25 (1.06–1.46)	1.60 (1.42–1.80)	<0.0001	
Serum TG ≥ 150 mg/dL	Ref.	1.12 (1.00–1.26)	1.27 (1.13–1.41)	1.22 (1.04–1.43)	1.33 (1.19–1.50)	<0.0001	
Serum HDL-C ≤ 40 mg/dL	Ref.	0.98 (0.86–1.11)	1.05 (0.93–1.19)	1.12 (0.94–1.33)	1.31 (1.16–1.49)	<0.0001	
BP ≥130/85 mmHg	Ref.	1.06 (0.94–1.20)	1.32 (1.18–1.49)	1.40 (1.18–1.67)	1.40 (1.24–1.58)	<0.0001	
Fasting glucose ≥100 mg/dL	Ref.	1.10 (0.98–1.24)	1.12 (1.00–1.26)	1.30 (1.11–1.52)	1.29 (1.15–1.46)	<0.0001	
Women							
BMI < 25 kg/m² n = 34441	*20,135*	*7205*	*4277*	*1026*	*1798*		
MetS	Ref.	1.33 (1.23–1.43)	1.52 (1.39–1.66)	1.42 (1.21–1.67)	1.79 (1.58–2.01)	<0.0001	0.0449
WC ≥ 80 cm	Ref.	1.39 (1.30–1.48)	1.41 (1.31–1.53)	1.29 (1.12–1.50)	1.71 (1.54–1.90)	<0.0001	0.0003
Serum TG ≥ 150 mg/dL	Ref.	1.14 (1.06–1.22)	1.30 (1.20–1.41)	1.20 (1.03–1.40)	1.38 (1.24–1.55)	<0.0001	0.5473
Serum HDL-C ≤ 50 mg/dL	Ref.	1.12 (1.06–1.19)	1.14 (1.06–1.23)	1.03 (0.90–1.19)	1.36 (1.23–1.50)	<0.0001	0.0477

BP ≥ 130/85 mmHg	Ref.	1.25 (1.18–1.33)	1.42 (1.32–1.53)	1.41 (1.24–1.62)	1.42 (1.28–1.57)	<0.0001	0.6113
Fasting glucose ≥ 100 mg/dL	Ref.	1.15 (1.06–1.24)	1.29 (1.18–1.41)	1.31 (1.11–1.54)	1.44 (1.27–1.62)	<0.0001	0.7439
BMI ≥ 25 kg/m² n = 13588	*5455*	*3215*	*2459*	*753*	*1706*		
MetS	Ref.	1.20 (1.10–1.32)	1.41 (1.28–1.55)	1.73 (1.48–2.03)	1.67 (1.49–1.87)	<0.0001	
WC ≥ 80 cm	Ref.	1.22 (1.09–1.37)	1.49 (1.30–1.70)	1.54 (1.23–1.94)	2.54 (2.10–3.06)	<0.0001	
Serum TG ≥ 150 mg/dL	Ref.	1.16 (1.05–1.27)	1.33 (1.20–1.47)	1.50 (1.28–1.76)	1.44 (1.28–1.61)	<0.0001	
Serum HDL-C ≤ 50 mg/dL	Ref.	1.14 (1.04–1.24)	1.14 (1.03–1.25)	1.40 (1.20–1.64)	1.30 (1.17–1.45)	<0.0001	
BP ≥ 130/85 mmHg	Ref.	1.22 (1.11–1.34)	1.33 (1.20–1.47)	1.46 (1.24–1.71)	1.50 (1.34–1.69)	<0.0001	
Fasting glucose ≥ 100 mg/dL	Ref.	1.18 (1.07–1.30)	1.28 (1.15–1.42)	1.53 (1.30–1.80)	1.50 (1.33–1.68)	<0.0001	

[a] ORs adjusted for: age (continuous), BMI (continuous), education (≤middle school, high school, ≥college, unknown), occupation (manual, non-manual, unemployed, unknown), marital status (married, single, unknown), smoking (never, past, current, unknown; only for men), alcohol drinking (current, non, unknown), regular exercise (yes, no, unknown), menopausal status (pre-, post-, unknown; only for women), and sleep duration (continuous). [b] MetS: the presence of 3 or more of the following components: 1) elevated waist circumference (WC); 2) high triglyceride (TG) levels; 3) low high density lipoprotein-cholesterol (HDL-C) or taking anticholesterol medication; 4) high blood pressure (BP) or taking antihypertensive medicine; 5) high fasting glucose levels or taking medication to treat diabetes mellitus. [c] Linear trends across snoring frequency categories were calculated by general linear regression. [d] BMI interaction term was assessed by likelihood ratio tests with the use of a cross-product term.

Table S2. Odds ratio (ORs) [a] of metabolic syndrome (MetS) [b] by number of obstructive sleep apnea (OSA) markers, the Health Examinees-Gem (HEXA-G) 2009–2013.

	Number of OSA Markers [c]				
	**None**	**1**	**2**	**3**	***p*-Trend** [d]
Men n = 24,856 [e]	*9447*	*9916*	*3904*	*1589*	
MetS	Ref.	1.51 (1.41–1.61)	1.86 (1.71–2.01)	2.03 (1.81–2.27)	<0.0001
WC ≥ 80 cm	Ref.	1.64 (1.53–1.75)	2.01 (1.85–2.18)	2.45 (2.19–2.74)	<0.0001
Serum TG ≥ 150 mg/dL	Ref.	1.31 (1.23–1.39)	1.45 (1.34–1.56)	1.53 (1.37–1.70)	<0.0001
Serum HDL-C ≤ 50 mg/dL	Ref.	1.10 (1.02–1.18)	1.02 (0.93–1.12)	1.17 (1.03–1.32)	<0.0001
BP ≥ 130/85 mm Hg	Ref.	1.32 (1.25–1.40)	1.59 (1.47–1.72)	1.62 (1.45–1.81)	<0.0001
Fasting glucose ≥ 100 mg/dL	Ref.	1.17 (1.10–1.24)	1.32 (1.22–1.43)	1.31 (1.18–1.47)	<0.0001
Women n = 48,029 [e]	*25,332*	*15,841*	*5858*	*998*	
MetS	Ref.	1.28 (1.21–1.35)	1.37 (1.27–1.47)	1.48 (1.28–1.72)	<0.0001
WC ≥ 80 cm	Ref.	1.26 (1.19–1.33)	1.12 (1.03–1.21)	1.09 (0.91–1.30)	<0.0001
Serum TG ≥ 150 mg/dL	Ref.	1.16 (1.10–1.21)	1.25 (1.17–1.33)	1.39 (1.21–1.60)	<0.0001
Serum HDL-C ≤ 50 mg/dL	Ref.	1.10 (1.05–1.15)	1.14 (1.07–1.21)	1.20 (1.05–1.37)	<0.0001
BP ≥ 130/85 mm Hg	Ref.	1.24 (1.19–1.30)	1.33 (1.25–1.41)	1.42 (1.24–1.63)	<0.0001
Fasting glucose ≥ 100 mg/dL	Ref.	1.17 (1.11–1.24)	1.27 (1.18–1.37)	1.38 (1.19–1.60)	<0.0001

[a] ORs adjusted for: age (continuous), BMI (continuous), education (≤middle school, high school, ≥college, unknown), occupation (manual, non–manual, unemployed, unknown), marital status (married, single, unknown), smoking (never, past, current, unknown; only for men), alcohol drinking (current, non, unknown), regular exercise (yes, no, unknown), menopausal status (pre-, post-, unknown; only for women), and sleep duration (continuous). [b] MetS: the presence of 3 or more of the following components: (1) elevated waist circumference (WC); (2) high triglyceride (TG) levels; (3) low high density lipoprotein-cholesterol (HDL-C) or taking anticholesterol medication; (4) high blood pressure (BP) or taking antihypertensive medicine; (5) high fasting glucose levels or taking medication to treat diabetes mellitus. [c] OSA markers include loud snoring, breathing interruptions and awakenings. [d] Linear trends across snoring frequency categories were calculated by general linear regression. [e] Gender *p*-interaction value < 0.0001; interaction term was assessed by likelihood ratio tests with the use of a cross-product term.

© 2017 by the authors; licensee MDPI, Basel, Switzerland. This article is an open access article distributed under the terms and conditions of the Creative Commons by Attribution (CC-BY) license (http://creativecommons.org/licenses/by/4.0/).
